# The Chinese pond mussel *Sinanodonta woodiana* demographically outperforms European native mussels

**DOI:** 10.1038/s41598-021-96568-1

**Published:** 2021-08-23

**Authors:** Maria Urbańska, Andrzej Kamocki, Małgorzata Kirschenstein, Małgorzata Ożgo

**Affiliations:** 1 Department of Zoology, Poznań University of Life Sciences, Wojska Polskiego 28, 60-637 Poznań, Poland; 2 Faculty of Civil Engineering and Environmental Sciences, Bialystok University of Technology, Wiejska 45 E, 15-351 Bialystok, Poland; 3 Institute of Navigation, Military University of Aviation, Dywizjonu 303 no. 35, 08-521 Dęblin, Poland; 4 Department of Evolutionary Biology, Kazimierz Wielki University, Ossolińskich 12, 85-093 Bydgoszcz, Poland

**Keywords:** Ecology, Conservation biology, Freshwater ecology, Invasive species, Ecology, Conservation biology, Freshwater ecology, Invasive species

## Abstract

Unionid mussels are essential for the integrity of freshwater ecosystems but show rapid worldwide declines. The large-sized, thermophilic Chinese pond mussel *Sinanodonta woodiana* *s.l.,* however, is a successful global invader, spread with commercially traded fish encysted with mussel larvae; its negative impacts on native mussels are expected. Here, we exploit a natural experiment provided by a simultaneous introduction of *S. woodiana* and four species of native unionids for water filtration to a pond in north-eastern Poland. *Sinanodonta woodiana* established a self-sustaining population and persisted for 19 years in suboptimal thermal conditions (mean annual temperature, 7.4 °C; mean temperature of the coldest month, − 3.7 °C, 73-day mean yearly ice-formation), extending the known limits of its cold tolerance. Over four study years, its frequency increased, and it showed higher potential for population growth than the native mussels, indicating possible future dominance shifts. Outbreaks of such sleeper populations are likely to be triggered by increasing temperatures. Additionally, our study documents the broad tolerance of *S. woodiana* concerning bottom sediments*.* It also points to the importance of intentional introductions of adult individuals and the bridgehead effect facilitating its further spread. We argue that *S. woodiana* should be urgently included in invasive species monitoring and management programmes.

## Introduction

Unionid mussels provide vital services in freshwater ecosystems by contributing to water purification, nutrient circulation, bottom bioturbation and provision of habitats^1^. They are, however, susceptible to environmental change due to several features of their biology: slow growth and late maturation, dependence on suitable host-fish for larval development, low dispersal abilities during adult life, and often narrow habitat specialisation^2,3^. Populations of unionid mussels decline rapidly throughout the world, and many are critically endangered^4,5^. Yet, some Asian pond mussels of the genus *Sinanodonta* are hyper-successful invaders^6^. Their expansion is associated with commercial trade in freshwater fish, which, when infested with mussel larvae (glochidia), serve as vectors for their spread^7–9^. Most notably, members of the Chinese pond mussel *Sinanodonta woodiana* species complex rapidly expand their range and have already colonised large parts of Europe and Russia, Southeast Asia and Australasia, Central America and the USA^8–12^. The lineage that invaded Europe originates from the Yangtze River basin^9,11^; the evolution of cold-tolerance through in situ adaptation has probably triggered its recently accelerated spread^11^.

*Sinanodonta woodiana* has several preadaptations for a rapid and successful invasion. It is a habitat generalist, inhabiting ponds, reservoirs, lakes, irrigation channels and rivers^13^, although possibly with a preference for sandy bottom substrates^14^. It utilises an extensive host range^15–17^ and has high growth and reproductive rates^17–19^. It is also tolerant to low water quality and pollution^20–23^.

**Figure 1:**
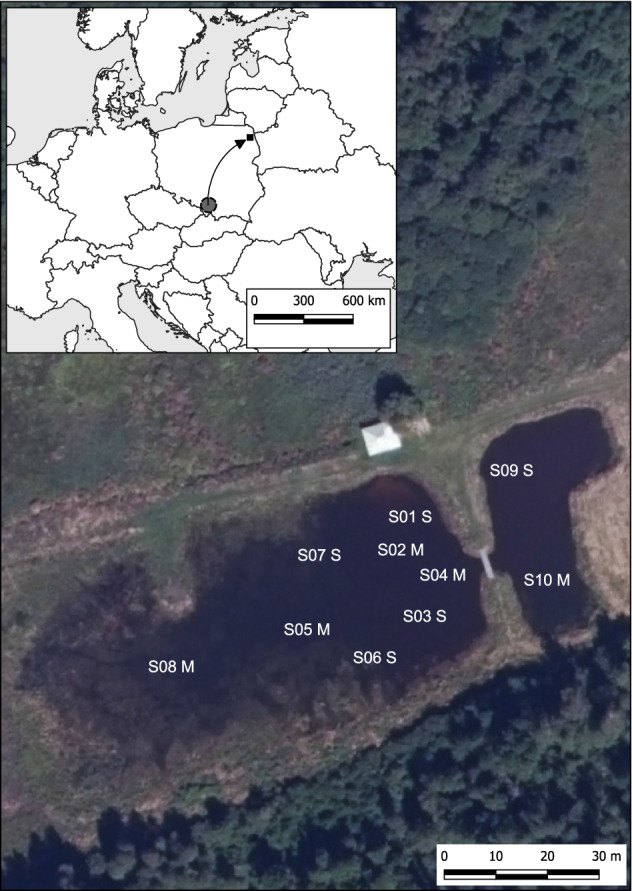
Map of the study area and location of the sampling sites. The grey circle indicates an approximate location of the source population for the introduction of *S. woodiana.* S01–S10, sampling sites; M, muddy bottom; S, sandy bottom. The map was generated using the QGIS 3.16.4-Hannover software (Free and Open Source Software (FOSS); Free Software Foundation, Inc., USA; www.qgis.org). Source for the orothophotomap: goeportal.gov.pl (Terms and conditions: https://www.geoportal.gov.pl/regulamin).

The ability of *S. woodiana* to outcompete native mussels has been predicted based on its exceptional body-size, with a shell length reaching over 25 cm and total wet body mass over 1.5 kg^7,24^, high filtration rates^25^, ability to induce cross-resistance in the host fish^26^, and a possible role in transmitting parasites and diseases^27,28^. Despite these predictions, the impacts of *S. woodiana* on the population dynamics of native mussels, as far as we know, have not been studied before. Such studies are usually hindered by the unknown time since introduction, the large spatial extent of the invaded habitats, their broad connectivity and inaccessibility. Here we exploit an exceptional opportunity provided by a natural experiment inadvertently set up in north-eastern Poland. In 2000, a pond fed by groundwater was made in the edge zone of a fen complex in the Biebrza River basin (N53.7517, E23.3102, Fig. 1) for water retention and recreation. In the same year, to improve water quality, the owners of the pond introduced native unionid mussels: *Anodonta anatina*, *A. cygnea*, *Unio pictorum* and *U. tumidus* originating from local populations. Additionally, several individuals (“a bucketful”) of *S. woodiana* were brought purposely from southern Poland (a straight-line distance of over 500 km) as this species is known to be particularly effective at water filtration. The area of origin of these individuals harbours well-established populations of *S. woodiana* ^29^. The pond was regularly stocked with fish: *Alburnus alburnus*, *Ctenopharyngodon idella, Cyprinus carpio*, *Esox lucius, Rutilus rutilus, Hypophthalmichthys molitrix,* *Perca fluviatilis,* and *Tinca tinca* originating from local sources; fish from hatcheries with heated water were not introduced. The pond is located in the coldest region of the combined natural and invasive range of established *S. woodiana* populations, excluding its presence in heated water effluents (climatic characteristics of the study area are given in the “Methods” section).

Following the discovery of this site in 2016, we carried out a comparative study of the structure and dynamics of the *S. woodiana* and native unionid populations over four consecutive years. We addressed the following hypotheses: (i) *S. woodiana* would persist and successfully reproduce despite the harsh climatic conditions; (ii) population structure would differ among species, with more dynamic recruitment in *S. woodiana* than the native mussels; (iii) spatial distribution would differ among species reflecting their habitat preferences, and *S. woodiana* would reach higher densities at sites with a sandy than a muddy bottom. Additionally, our study points to intentional introductions of adult *S. woodiana* individuals as an important route of dispersal of this invasive species.

## Results

### Species composition and changes in relative frequencies

**Figure 2:**
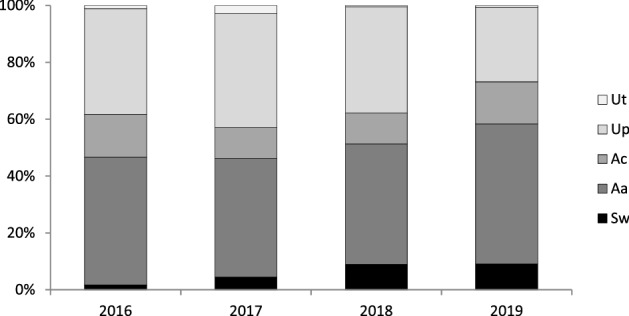
Relative frequencies of the co-occurring unionid mussels in 2016–2019. Ut, *Unio tumidus*; Up, *U. pictorum*; Ac, *Anodonta cygnea*; Aa, *A. anatina*; Sw, *Sinanodonta woodiana*. N_2016_ = 350, N_2017_ = 1198, N_2018_ = 1058, N_2019_ = 596.

*Sinanodonta woodiana* co-occurred with native unionids: *Anodonta anatina*, *A. cygnea*, *Unio pictorum,* and *U. tumidus*. No other invasive bivalves were observed. *Anodonta anatina* was a dominant species, with a frequency ranging between 42 and 49%. The frequency of *A. cygnea* ranged between 11 and 15%, and *U. pictorum* between 26 and 40%. The frequency of *U. tumidus* did not exceed 3% in any of the study years. The frequency of *S. woodiana* was 1.7% in 2016, 4.5% in 2017, 9.0% in 2018, and 9.1% in 2019. The increase in its frequency over time was statistically significant (χ^2^_12_ = 99.3, *P* < 0.001; Fisher’s exact test, *P* < 0.008, Fig. 2).

### Reproductive status of *S. woodiana*

Of the 37 *S. woodiana* individuals dissected in July 2018, 16 were males, and 21 were females. Glochidia at various stages of maturation were found in 17 of the females.

### Shell-length distributions

**Figure 3:**
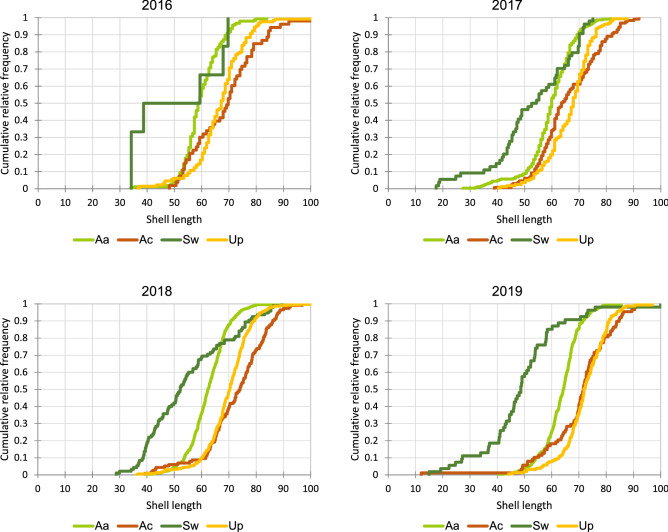
Cumulative shell-length distributions in *S. woodiana* and co-occurring native mussels in 2016–2019 (measurements min–max normalised). Aa, *Anodonta anatina* (N_2016_ = 157, N_2017_ = 245, N_2018_ = 448, N_2019_ = 294); Ac, *A. cygnea* (N_2016_ = 53, N_2017_ = 129, N_2018_ = 115, N_2019_ = 88); Sw, *Sinanodonta woodiana* (N_2016_ = 6, N_2017_ = 54, N_2018_ = 95, N_2019_ = 54); Up , *Unio pictorum* (N_2016_ = 130, N_2017_ = 161, N_2018_ = 395, N_2019_ = 156).

Consistently over the study period, smaller-sized mussels contributed a higher proportion of individuals in *S. woodiana* than in the native mussels (Fig. 3). Except for 2016, when the *S. woodiana* sample consisted of only six individuals, differences in the shell-length distribution between *S. woodiana* and the native mussels were statistically significant (Kolmogorov–Smirnov test, *P* < 0.0001 in all comparisons, Bonferroni corrected significance level *P* = 0.0083). In *A. anatina* and *U. pictorum*, mean shell-lengths and shell-length distributions changed over time towards an increasing dominance of larger individuals, with statistically significant differences between the consecutive years (Tables 1 and 2). In *A. cygnea*, statistically significant changes towards the dominance of larger-sized mussels were observed between 2017 and 2018 and between 2017 and 2019. In *S. woodiana*, changes in shell-size distribution between study years were not statistically significant despite the annual removal of collected individuals, carried out in adherence to the general guidelines on dealing with invasive species. Individual shell-length measurements are given in Supplementary Table S1 online.

**Table 1:** Summary statistics of shell length in co-occurring mussel populations in 2016—2019.

Species	Study year	Mean ± SD [mm]	Range [mm]	N
*A. anatina*	2016 A	89.9 ± 10.6	51–126	157
	2017 A	89.6 ± 13.3	41–131	245
	2018 B	93.7 ± 10.8	58.8–150.1	448
	2019 C	95.5 ± 10.2	60.8–128.4	294
*A. cygnea*	2016 A, B	111.1 ± 19.3	78–162	53
	2017 A	107.1 ± 17.9	63–149	129
	2018 B	117.6 ± 18.8	63.4–157.0	115
	2019 B	114.0 ± 19.8	19.6–155.9	88
*U. pictorum*	2016 A	87.3 ± 12.3	48–131	130
	2017 A	87.1 ± 11.0	53–115	161
	2018 B	91.3 ± 11.2	47.5–131.1	395
	2019 C	94.4 ± 10.9	57.9–127.1	156
*S. woodiana*	2016 A	112.7 ± 37.5	76–155	6
	2017 A	117.6 ± 33.4	39–167	54
	2018 A	122.4 ± 33.6	63.9–199.0	95
	2019 A	108.3 ± 32.8	33.3–225.5	54

Letters A, B, C denote significant differences in the Kruskal–Wallis test with Dunn’s pairwise comparison, Bonferroni corrected significance level *P* = 0.0083.

**Table 2:** P-values in the Kolmogorov–Smirnov test on changes in the cumulative shell-length distributions over the study period (measurements min–max normalised); Bonferroni corrected significance level, *P* = 0.0083.

*A. anatina*	2017	2018	2019
2016	> 0.05	< 0.0001	< 0.0001
2017		= 0.0001	< 0.0001
2018			= 0.0080

*A. cygnea*	2017	2018	2019
2016	> 0.05	= 0.048	> 0.05
2017		< 0.0001	< 0.0001
2018			> 0.05

*U. pictorum*	2017	2018	2019
2016	> 0.05	= 0.0003	< 0.0001
2017		= 0.0015	< 0.0001
2018			= 0.028

*S. woodiana*	2017	2018	2019
2016	> 0.05	> 0.05	> 0.05
2017		> 0.05	> 0.05
2018			> 0.05

### Species distribution and densities in relation to bottom sediments

*Sinanodonta woodiana, A. anatina*, *A. cygnea*, and *U. pictorum* were present at all sampling sites, and within sites, no clustering or spatial separation among species was observed. Summary statistics of mussel densities at sites differing in the bottom substrate are given in Table 3. Overall mussel densities did not differ significantly between the study years (two-way ANOVA, F_3_ = 1.43, *P* = 0.24) or bottom types (F_1_ = 0.39, *P* = 0.53), but there were significant differences among species (F_3_ = 14.00, *P* < 0.0001). Interaction between species and bottom type also had a significant effect (F_3_ = 9.54, *P* < 0.0001). The bottom type had a significant effect in *A. cygnea* (F_1_ = 14.67, *P* < 0.001) and *U. pictorum* (F_1_ = 9.56, *P* < 0.01): in *A. cygnea*, densities were higher at sites with a muddy bottom, and in *U. pictorum* at sites with a sandy bottom (Tamhane’s T2 pairwise comparisons, *P* < 0.001 and *P* < 0.01, respectively). The effect of bottom type was not significant in *A. anatina* (F_1_ = 1.40, *P* = 0.25) and *S. woodiana* (F_1_ = 0.19, *P* = 0.67), although both species occurred at higher densities at sites with a sandy bottom. Year and interaction between bottom type and year did not have a significant effect in any of the species.

**Table 3:** Summary statistics of mussel densities at sites with muddy (M) and sandy (S) bottom.

Species	Study year	M		S	
		Mean ± SD [ind. m^−2^]	Range [ind. m^−2^]	Mean ± SD [ind. m^−2^]	Range [ind. m^−2^]
*A. anatina*	2016	0.87 ± 0.72	0.40–1.70	2.02 ± 1.26	0.45–3.50
	2017	1.77 ± 1.25	0.15–3.60	4.40 ± 4.22	0.35–11.30
	2018	3.14 ± 1.76	1.70–6.10	2.78 ± 1.40	1.10–4.10
	2019	2.79 ± 1.80	0.60–5.00	2.57 ± 1.59	1.55–4.40
*A. cygnea*	2016	1.22 ± 0.67	0.45–1.70	0.89 ± 0.11	0.05–0.30
	2017	1.11 ± 0.82	0.00–2.10	0.63 ± 0.57	0.05–1.55
	2018	1.67 ± 1.26	0.20–3.60	0.13 ± 0.17	0.00–0.40
	2019	1.50 ± 1.52	0.30–3.70	0.35 ± 0.35	0.00–0.70
*U. pictorum*	2016	0.10 ± 0.17	0.00–0.30	1.60 ± 1.59	0.10–3.85
	2017	0.03 ± 0.04	0.00–0.10	4.91 ± 4.46	1.30–11.75
	2018	0.56 ± 0.42	0.10–1.20	3.70 ± 2.40	1.10–6.30
	2019	1.32 ± 1.42	0.10–3.30	1.17 ± 0.71	0.40–1.80
*S. woodiana*	2016	0.07 ± 0.12	0.00–0.20	0.08 ± 0.10	0.00–0.20
	2017	0.18 ± 0.14	0.00–0.30	0.46 ± 0.17	0.20–0.65
	2018	0.52 ± 0.39	0.15–1.05	0.58 ± 0.40	0.10–1.10
	2019	0.55 ± 0.56	0.00–1.30	0.53 ± 0.28	0.25–0.80

Number of sites with a muddy bottom, 2016: 3, 2017: 5, 2018: 5, 2019: 4. Number of sites with a sandy bottom, 2016: 4, 2017: 5, 2018: 5, 2019: 3.

### Observations on the behaviour of *S. woodiana* individuals

This study provided an opportunity for preliminary observations on the behaviour of *S. woodiana.* Compared to the native unionids, *S. woodiana* was often burrowing much deeper into the sediments, and some individuals were found 20 cm beneath the surface of the sandy substrates. At a low water level during the hot and dry summer in 2018, we observed *S. woodiana* individuals crawling over distances of up to 10 m in one day.

## Discussion

This study contributes to the understanding of the population dynamics of *S. woodiana* and its native counterparts during the early stages of invasion. It documents a self-sustaining population of *S. woodiana* in an area with cold and long winters and extends the known limits of its thermal tolerance. Comparison of demographic profiles shows a more favourable population structure in *S. woodiana* than in the native mussels, indicating possible future dominance shifts. This study also shows that *S. woodiana* is a habitat generalist concerning bottom sediments, and points to intentional introductions of adult individuals as an important and underappreciated route of dispersal of this invasive species.

### Thermal tolerance of *S. woodiana*

The introduction of *Sinanodonta woodiana* in 2000 resulted in a long-term establishment of its reproducing population, as evidenced by a high proportion of females carrying glochidia (17 out of 21 in 2018) and the presence of juveniles (the smallest individual of 33 mm shell-length was collected in 2019). While the populations of native mussels might have been augmented with glochidia attached to the stocking fishes, this was less likely in *S. woodiana*. The species was not recorded in the vicinity before^13^, and given the distance of over 500 km over which the founding individuals were transported, their local availability was unlikely. In any case, as the stocking fishes originated only from local sources, which did not include heated-water hatcheries, any *S. woodiana* glochidia would have also been from locally-adapted populations.

Winters in the study area are relatively cold and long. In 2000–2019, the mean temperature of the coldest month was − 3.7 °C, and the lowest mean monthly temperature was − 10.8 °C. The absolute minimum temperature was − 31.1 °C. Ice formed each year, on average for 73 days, with a maximum of 104 days. As far as we know, these are the most extreme climatic conditions in which an established population of this species was documented to date. *Sinanodonta woodiana* has been reported from Sweden, but no reproduction was observed there^30^. The populations in the Yenisei and Ob River basins inhabit heated water effluents^9,31^. The other population in northern Poland in a thermally-unpolluted water body is in a milder climate^24^. Thus, our study extends the known limits of cold tolerance of *S. woodiana*, indicating a shift in its realized niche^32^ or an ongoing in situ adaptation^11^.

Furthermore, with the ongoing climate change, the abiotic conditions in the invaded range of *S. woodiana* increasingly match its physiological optimum^33^. In our study area during the time since mussel introduction, the mean annual temperature increased by 0.8 °C, the number of days with ice formation decreased by 21, and the number of days with temperatures over 15 °C (coinciding with the production of ripe glochidia by *S. woodiana*^15^) increased by nine. *Sinanodonta woodiana* survives at water temperatures up to 38 °C^34^ and has a higher tolerance to thermal stress than the native mussels^21^. In heated water bodies it reproduces throughout the year^18^ and occupies habitats with higher temperature ranges than the native unionids^14^, indicating that climate warming will increase its competitive advantage. Additionally, high mobility of *S. woodiana* and its tendency to burrow deeply into the sediments may help it better survive during drought episodes.

As shown in our study, in suboptimal thermal conditions, *S. woodiana* can persist at low abundances for decades. Outbreaks of such sleeper populations (sensu^35^) are likely to be triggered by changes in the environment, e.g., rising temperatures.

### Population structure of *S. woodiana* in relation to native unionids

Over four study years, the relative frequency of *S. woodiana* increased from 2 to 9%. A comparison of shell-length distributions, approximating population age-structure, shows that smaller-sized mussels contributed a higher proportion of individuals in *S. woodiana* than in the native mussels in all study years, and this difference was increasing over time. This increase over time was possibly related to the removal of *S. woodiana* individuals, as hand-sampling tends to be biased towards larger individuals. On the other hand, the high mobility of this species and its striking burrowing behaviour, which lowers its detectability, might have counterbalanced this effect, as illustrated by the largest *S. woodiana* individual, with a shell length of 22.5 cm, found in the last study year. Nevertheless, it is possible that without the removal of individuals, the size structure would also shift towards larger sizes in *S. woodiana*, and its relative abundance at the study sites would increase even faster. Interestingly, a higher contribution of smaller-sized individuals in *S. woodiana* than in the native mussels was also observed in^24^, where no mussels were removed before the study. Thus, in both these studies, *S. woodiana* not only established viable populations but also showed higher potential for population growth than the native mussels. This is not surprising given that *S. woodiana* grows faster, matures earlier and produces more glochidia per female than the native unionids^17–19,36^. At increasing relative frequencies, its direct effects on the native unionids: competition for food, bottom space and host fish, filtering out sperm and larvae, and transmission of diseases^6^ will play an increasing role, and a dominance shift can be expected. This, in turn, is expected to affect ecosystem functioning, including changes in water transparency and nutrient availability^25,37^, benthic habitat modification^38,39^, and reduction in the condition of fish^40^. Additionally, *S. woodiana* invasion threatens the endangered European bitterling *Rhodeus amarus*^16^, and its massive die-offs negatively impact water quality and reverberate to terrestrial ecosystems^41,42^.

The increasing prevalence of *S. woodiana* in invaded areas^17,23,43,44^ supports its predicted ability to effectively compete with native mussels. Our present study shows that demographic profiles of co-occurring mussel populations can indicate future dominance shifts already at initial invasion stages. However, as in many alien species^45,46^, the time-lag between the establishment of *S. woodiana* and the expression of its impacts can last decades, explaining why, despite its striking body-size (“a football-sized invasive mussel”^47^), the threats from its invasion are largely underestimated.

### Tolerance of *S. woodiana* for bottom sediment type

Despite a large number of studies documenting the spread of *S. woodiana* (for a recent summary, see, e.g.,^11,48^), not much is known on its preferences concerning bottom sediments. *Sinanodonta woodiana* is mainly reported from ponds and reservoirs, which suggests its preference for muddy sediments. However, its presence in these habitats is related to its mode of dispersal rather than habitat preferences. Basing on a study in a heated lakes system with various habitats, Kraszewski and Zdanowski^14^ suggested a preference of *S. woodiana* for sandy bottom substrates. The patchy distribution of sandy and muddy bottom substrates allowed us to test this hypothesis in the present study.

According to expectations, based on the known preferences of the native species^49^, *A. cygnea* occurred predominantly at sites with a muddy bottom, *U. pictorum* at sites with a sandy bottom, and *A. anatina* occurred at similar densities on both bottom types. Contrary to expectations, however, *S. woodiana* did not show a preference for either bottom type. Although its overall density was higher at sites with a sandy than a muddy bottom, this difference was not significant. *Sinanodonta woodiana* can utilize a broad range of host-fish species^15–17^ and survive in a broad range of water-body types^13^. Our study indicates that it is also a habitat generalist concerning bottom sediments and adds to the suit of the known tolerances of this species.

### Intentional human-mediated dispersal

The global spread of *S. woodiana* is primarily due to the trade in freshwater fish^7,9^. Our study points to intentional introductions for water filtration as an additional route of dispersal of this species. Large individual sizes and arguably beautiful colouration of *S. woodiana* add to its perceived attractiveness, and some people are willing to undertake considerable efforts to obtain individuals of this species. Occasional long-distance translocations can cause the bridgehead effect^46,50^, in which the establishment of populations in new locations facilitates the further dispersal of the species and leads to a self-accelerating invasion process. The way humans interact with invasive species is one of the main determinants of their spread and establishment^51,52^. Our local interviews indicate that individuals from the study pond have already been transferred to nearby water bodies, and their filtering ability is highly appreciated. The propensity of people to acquire and translocate *Sinanodonta* mussels has been noted before^13,17,24,53–55^ and is probably more important than previously appreciated.

### Management implications

Eradication of established invasive bivalve populations is extremely difficult^6^. An apparently successful attempt to eradicate *S. woodiana* from invaded fish ponds involved lowering the water level and poisoning the fish and mussels^10,47^, but usually such measures cannot be applied. An alternative is the removal of individuals by hand harvesting. To be effective, however, it should cover the whole surface of the invaded water body and be repeated regularly. A related, commonly used practice in field research on invasive species is to remove the collected individuals from the study area. Our study shows that at least in *S. woodiana*, this is not likely to have any practical effect. We took out all individuals collected during four annual surveys from collection sites covering approximately 8% of the surface area of the pond. The relative frequency of *S. woodiana* increased while its densities and shell-length distributions remained unchanged. This was not unexpected, given a small proportion of the population sampled, combined with the high reproduction rates and mobility of this species. As sampling rarely includes more than 10% of the studied populations, alternatively to removing individuals from a study area, long-term studies involving marking and releasing them back might be considered. Knowledge of the biology of *S. woodiana* in the wild (e.g., growth rates, longevity, behavioural responses) is scarce, limiting our ability to manage and reduce its further spread.

The priority, however, is to prevent introductions of *S. woodiana* to non-invaded water bodies. Fish trade remains its dominant dispersal route, so effective biosecurity measures are necessary. Well-coordinated monitoring programmes are needed for evidence-based management decisions^56^. Public participation is key to successful management of invasive species. Publicly accessible educational programmes explaining the problems of invasive species and increasing the appreciation of the native ones are required, especially when the invasive species elicit favourable reactions from people^51^, as is the case with *S. woodiana*.

*Sinanodonta woodiana* does not yet have the status of a recognized pest. For example, it is not included in the list of invasive alien species of European Union concern^57^ and there are no regulations concerning this species in most countries. Our study documents the potential of *S. woodiana* to demographically outcompete native unionids. Combined with its recognized impacts and rates of spread, it highlights the need to urgently call the attention of policymakers and the public to the threats posed by *S. woodiana* to the integrity of freshwater ecosystems.

## Conclusions

This study documents the potential of *S. woodiana* for long-term persistence at low abundances in suboptimal thermal conditions. *Sinanodonta woodiana* established a self-sustaining population and persisted for almost 20 years in an area with cold and long winters. Outbreaks of such sleeper populations (sensu^35^) are likely under the rising temperatures scenario. Over four study years, the frequency of *S. woodiana* increased from 2 to 9%. Already at this low-abundance invasion stage, it showed a more favourable population structure than the co-occurring native mussels, indicating possible future dominance shifts. The tolerance of *S. woodiana* concerning bottom substrates adds to the suit of the broad tolerances of this species that contribute to its invasive potential.

*Sinanodonta woodiana* is usually spread with fish infested with its larvae. In this study, several adult individuals were transported from over 500 km away, purposely introduced for water filtration and subsequently translocated to other water bodies in the vicinity. This illustrates the importance of how people interact with invasive species and points to the underappreciated role of intentional introductions of adult *S. woodiana* that can lead to the bridgehead effect facilitating its further spread.

The potential of *S. woodiana* to demographically outcompete native mussels, combined with its recognized impacts and rates of spread, justify its urgent inclusion in monitoring and management programmes. Publically accessible educational programmes are needed to increase the awareness of the problems produced by this species and an appreciation of native bivalves.

## Methods

### Climatic conditions

**Figure 4:**
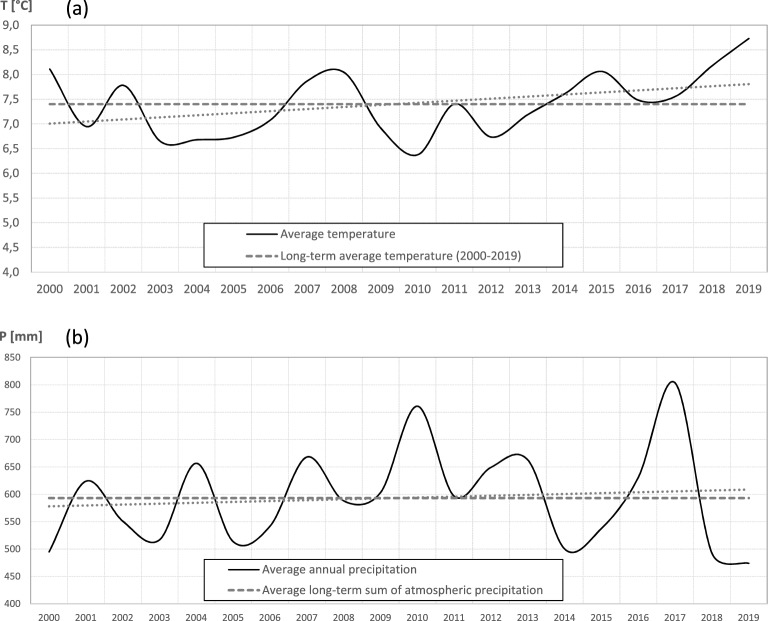
Variation in the mean annual temperature (**a**) and mean annual precipitation (**b**) in the study area in 2000–2019.

To characterise climatic conditions, we used temperature and precipitation records provided by the Polish Institute of Meteorology and Water Management collected at the meteorological stations in Biebrza and Suwałki, which lie at a straight-line distance of 50 and 49 km from the study area within the same isotherm values. In 2000–2019, i.e. during the time since the introduction of *S. woodiana* till the end of our study, the mean (± SD) ambient temperature was 7.4 ± 0.6 °C, ranging from 6.4 °C (2010) to 8.7 °C (2019), (Fig. 4). The mean temperatures of January and July were − 3.7 ± 2.9 °C and 18.5 ± 1.5 °C, respectively. The lowest mean monthly temperature was − 10.8 °C (January 2010) and the highest 21.1 °C (July 2010). The absolute minimum temperature was − 31.1 °C (7 January 2003), and the absolute maximum temperature was 34.2 °C (8 August 2010). The number of days with ice cover (mean daily temperature below 0.0 °C) was 73 ± 18 per year, ranging from 45 (2019) to 104 (2010). The number of days with mean daily temperatures exceeding 15.0 °C and 20 °C was 87 ± 11 and 22 ± 9, respectively. Linear trends indicate that over 2000–2019, the mean annual temperature increased by 0.8 °C, the mean number of days with ice formation decreased by 21, and the mean number of days with daily mean temperatures exceeding 15.0 °C and 20 °C increased by 9 and 6 days, respectively.

The annual sum of precipitation was 593 ± 86 mm, ranging from 474 mm (2019) to 803 mm (2017). Periods with low rainfall coincided with periods with high temperatures resulting in drought episodes, with an increasing tendency in frequency.

### Physicochemical water parameters

Physicochemical water parameters (pH, specific conductance, and dissolved oxygen) were measured in June 2017 in the field with a Hach Lange HQ40D probe (Hach, Germany) at 12 sites in different parts of the pond. The other parameters were determined in the laboratory in three water samples: the content of phosphorus and nitrogen was measured using UV-1800 spectrophotometer (Shimadzu, Japan); total carbon was determined in TOC-L analyser with SSM-5000A Solid Sample Combustion Unit (Shimadzu, Japan); the concentrations of Ca and Mg were determined by atomic absorption spectroscopy (Avanta AAS, GBC Scientific Equipment Ltd.).

The mean (± SD) pH was 7.84 ± 0.08, the mean specific conductance was 393 ± 30.2 µS cm^−1^, and the mean dissolved oxygen content was 5.5 ± 0.8 mg dm^3^. The concentration of N-NO_3_ was 0.64 ± 0.07 mg dm^3^, of N-NH_4_ was 0.42 ± 0.12 mg·dm^−3^, and of P-PO_4_^3−^ was below the quantification limit (< 0.05 mg dm^3^). The concentration of dissolved organic carbon (TOC) did not exceed 40 mg·dm^−3^. The concentrations of Ca^2+^ and Mg^2+^ were 64.6 ± 16.1 mg dm^3^ and 14.7 ± 1.0 mg dm^3^, respectively.

### Mussel collection and documentation

During the study period, the bottom of the pond consisted of interspaced sandy and muddy patches. In 2016 we established seven sampling sites: four with a sandy bottom and three with a mud layer of approximately 30 cm. In 2017 and 2018, we included three additional sites (together, 5 with a sandy bottom and 5 with a muddy bottom). In 2019, parts of the pond dried out during a hot and dry summer, and sampling was possible at only three sandy sites and four muddy sites. Mussels were hand-collected by wading, snorkelling or scuba diving, depending on water depth, from areas delineated with metal chains placed on the bottom. The collection area covered approximately 30% of a patch with a uniform bottom substrate (20 m^2^ at sites S01, S05, S06, S07, S08, S09, and 10 m^2^ at sites S02, S03, S04, S10).

In unionid mussels, age determination based on external growth annuli is often unreliable^58^ and was ambiguous in our study area, so we used shell-length distributions to approximate the age-structure of the populations. We measured shell-lengths of all individuals to the nearest 1 mm in 2016 and 2017 and the nearest 0.1 mm in 2018 and 2019. In 2017, due to adverse weather conditions, we measured only 30 randomly chosen individuals from the largest subsamples of *A. anatina* (collected at sites S01, S03, S09) and *U. pictorum* (S01, S07, S09); together in 2017, we measured 245 individuals of *A. anatina* out of collected 500, and 161 individuals of *U. pictorum* out of 480. We released the native mussels at sites of collection. In adherence to the commonly adopted practice in research on invasive species, we removed the collected *S. woodiana* from the study area. In 2018, we dissected 37 individuals of this species to determine their reproductive status. We used the opportunities provided by this study to document some observations on the behaviour of *S. woodiana*. We estimated the distance covered by individual mussels by measuring the length of the traces left on the sediment surface, clearly visible at a low water level during the hot and dry summer in 2018. On observing the striking burrowing behaviour of some *S. woodiana* individuals, we measured the depth at which they were found with a ruler.

### Data analysis

We included all mussel species in the analysis of the relative frequencies. In the analyses of shell-length distributions and mussel densities, we omitted *U. tumidus*, which occurred in low numbers throughout the study period. Changes in relative frequencies were tested with the χ^2^ homogeneity test and Fisher’s exact test. Differences in mean shell lengths between study years were compared using the Kruskal–Wallis test followed by Dunn’s pairwise comparisons with a Bonferroni corrected significance level. Differences in cumulative shell-length distributions between species and study years were compared using the Kolmogorov–Smirnov test with Bonferroni-corrected significance level. To account for shell-size differences among species, we rescaled shell-length measurements from 0 to 100 in each species over measurements from the whole study period using min–max normalisation. Because of a generally low detectability of small mussels during hand-sampling, we set a minimum shell-length at 0 mm in each species to avoid accidental differences among them; we omitted this value in the analyses and graphical presentations. Differences in mussel densities were tested using the two-way analysis of variance (ANOVA) with species, bottom type and study year as explanatory variables, followed by Tamhane’s T2 multiple pairwise comparisons. Density data were log_10_ transformed to meet assumptions of normality. All analyses were carried out with XLStat 2020.

## Supplementary Information

Supplementary Information.

## Data Availability

The datasets generated and analysed during this study are included in this published article and its Supplementary Information files.
